# On visual hallucinations and cortical networks: a trans-diagnostic review

**DOI:** 10.1007/s00415-015-7687-6

**Published:** 2015-03-13

**Authors:** Rowena Carter, Dominic H. ffytche

**Affiliations:** 1 Academic Clinical Fellow in General Psychiatry, King’s College London, London, UK; 2 South London and Maudsley NHS Foundation Trust, London, UK; 3 Institute of Psychiatry, Psychology and Neuroscience, King’s College London, London, UK

**Keywords:** Parkinson’s disease, Alzheimer’s disease, Dementia with Lewy bodies, Schizophrenia, MRI, Structural imaging, Grey matter

## Abstract

Our current clinical approach to visual hallucinations is largely derived from work carried out by Georges de Morsier in the 1930s. Now, almost a century after his influential papers, we have the research tools to further explore the ideas he put forward. In this review, we address de Morsier’s proposal that visual hallucinations in all clinical conditions have a similar neurological mechanism by comparing structural imaging studies of susceptibility to visual hallucinations in Parkinson’s disease, Alzheimer’s disease, Dementia with Lewy bodies and schizophrenia. Systematic review of the literature was undertaken using PubMed searches. A total of 18 studies across conditions were identified reporting grey matter differences between patients with and without visual hallucinations. Grey matter changes were categorised into brain regions relevant to current theories of visual hallucinations. The distribution of cortical atrophy supports de Morsier’s premise that visual hallucinations are invariably linked to aberrant activity within visual thalamo-cortical networks. Further work is required to determine by what mechanism these networks become predisposed to spontaneous activation, and whether the frontal lobe and hippocampal changes identified are present in all conditions. The findings have implications for the development of effective treatments for visual hallucinations.

## Introduction

In 1930, Georges de Morsier published a critique of the widely accepted psychodynamic account of hallucinations, suggesting instead they be considered as neurological symptoms [1]. He had been studying in Paris under de Clérambault who, influenced by studies of epilepsy, developed a theory of psychosis in which its constituent features were caused by aberrant neural activity within specific brain networks leading to psychopathological symptoms (mental automatisms) [2]. Central to this view was the assumption that the location of the aberrant circuit for a given symptom was invariant across clinical conditions. de Morsier went on to apply these ideas to visual hallucinations—visual automatisms in his terms—reviewing their similarity across neurological and psychiatric contexts as evidence for a mechanistic cause.

….existe-t-il une différence entre les automatismes ou hallucinations visuelles accompagnant les syndromes neurologiques…..et les automatismes ou hallucinations visuelles qu’on rencontre dans les psychoses hallucinatoires chroniques? Nous sommes fondés à répondre qu’il n’en existe aucune. Les unes comme les autres peuvent avoir les mêmes caractères et apparaissent de façon semblable parce que les unes comme les autres sont d’origine strictement mécanique [3]. (…is there a difference between automatisms or visual hallucinations accompanying neurological syndromes…and automatisms or visual hallucinations encountered in chronic hallucinatory psychosis? We are led to reply that there is none. One like the other can have the same features and appear in a similar way because one like the other is of strictly mechanical origin).

**Fig. 1:**
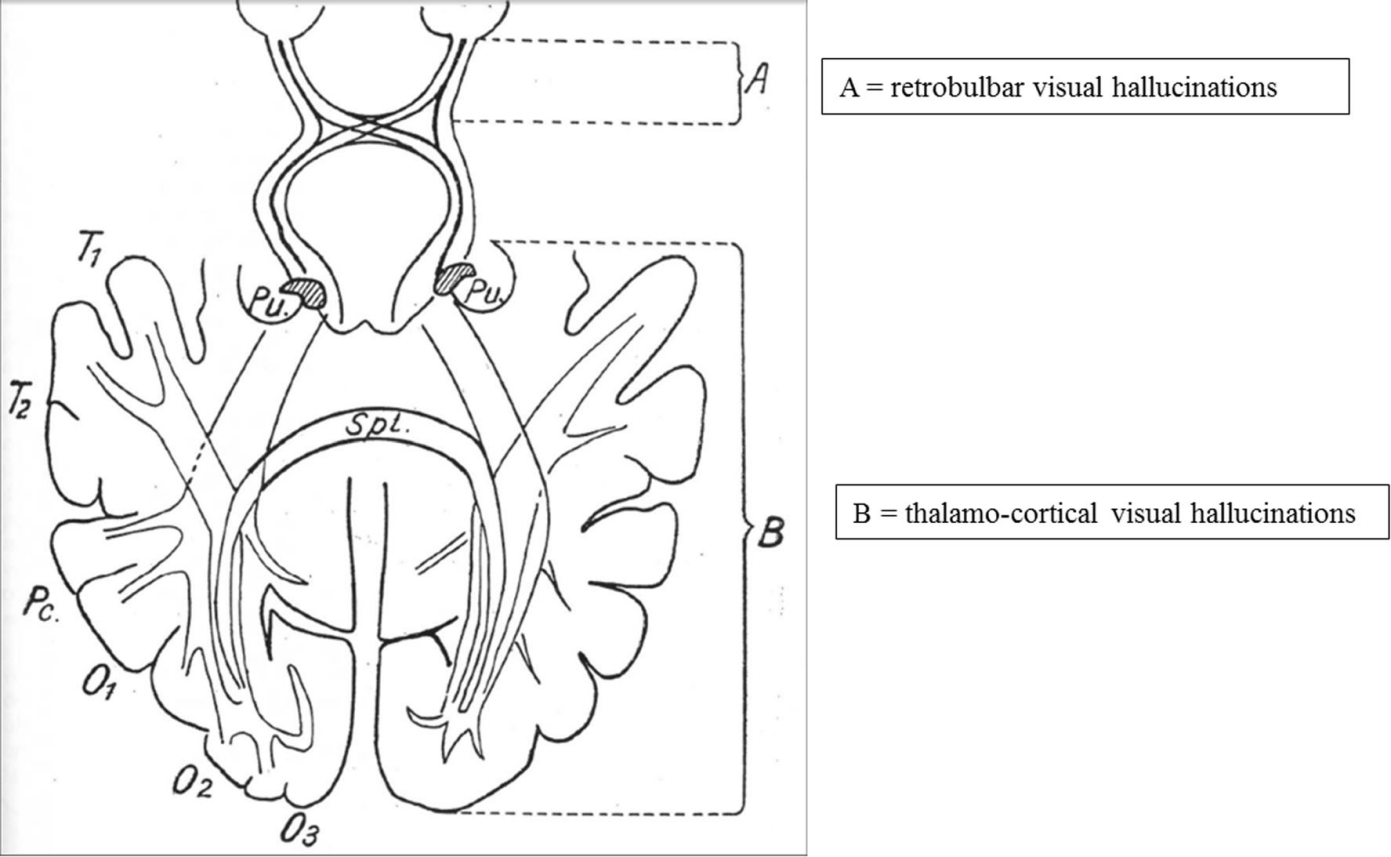
The anatomical pathways linked to visual hallucinations as proposed by de Morsier. Hallucinations seen in both eyes were linked to thalamo-cortical networks connecting either the lateral geniculate nucleus and primary visual cortex or pulvinar and partieto-occipital cortex (**b**). Hallucinations seen in one eye were linked to the anterior visual pathways (**a**). Adapted from [4]

For de Morsier, apart from rare visual hallucinations associated with one or other eye which he linked to anterior visual pathways, the predominant underlying cause of visual hallucinations was aberrant activity in thalamo-cortical networks connecting either the lateral geniculate nucleus to primary visual cortex or pulvinar to partieto-occipital cortex (Fig. 1) [3, 4]. Yet, while confident about the brain location of this aberrant activity, he recognised that research techniques were not yet available to investigate their underlying neurophysiology [1].

Now, approaching a century after these ideas were first formulated, we have the research tools to begin to address them. At one level, de Morsier seems to have been correct. Based on the evidence of functional imaging, at the time a visual hallucination is experienced spontaneous ‘aberrant’ increases in activity occur within the visual system. This is the case, irrespective of whether the hallucination occurs in the context of eye disease [5] or schizophrenia/first episode psychosis [6, 7]. Note that a recent case study of Parkinson’s disease found suppression of activity in visual cortex at the time of visual hallucinations [8] which, if replicated, would point to a variation in underlying neurophysiology. However, even if activity increases turn out to be the rule, the functional imaging evidence only captures the end point of a longer causal chain. There are likely to be many different ways in which a given region of cortex becomes susceptible to spontaneous ‘aberrant’ activity. It might, for example, result from alterations in intra-cortical connections within a cortical region. Alternatively, it might result from changes in connectivity across a wider cortical and subcortical network. This deeper level of understanding of the neurophysiological cause of visual hallucinations was purely of academic interest for de Morsier as there were limited options for treatment. Today, the question of what predisposes a region of cortex to spontaneous activity and visual hallucinations is central to our understanding of how to treat them as different types of predisposing mechanism are likely to require different treatment approaches. de Morsier’s unanswered neurophysiological question might therefore be reformulated in the era of neuroimaging into one of whether the same changes in cortical networks are found in patients susceptible to visual hallucinations, irrespective of the clinical condition in which they occur.

In this review, we address this issue by comparing structural imaging studies of susceptibility to visual hallucinations in Parkinson’s disease, Alzheimer’s disease, Dementia with Lewy bodies, schizophrenia and eye disease (Charles Bonnet Syndrome). We focus on studies reporting grey matter differences between patients with and without visual hallucinations as the limited number of white matter, tractography and functional activation studies limits comparison across conditions. Most of the studies reviewed do not provide detailed cortical locations, limiting us to a qualitative summary in which we divide the brain into regions defined by their hypothesised role in current accounts of visual hallucinations.

## Methods

The imaging studies were identified from citations in expert reviews and PubMed searches using combinations of keywords ‘visual hallucinations’, ‘structural imaging’ and ‘MRI’ for each of the conditions. After excluding studies of visual hallucinations in the context of delirium or a single occurrence, a total of 18 studies were identified reporting grey matter differences between patients with and without visual hallucinations. The grey matter changes reported in each study were categorised into (i) occipital cortex including primary visual cortex (grey matter changes encompassing calcarine cortex on the medial occipital lobe) and visual association cortex (regions in the lateral, ventral and dorsal occipital lobe); (ii) superior and inferior parietal lobules to include the dorsal visual pathway, parietal eye fields and supra-marginal gyrus; (iii) the temporal lobe to include the ventral visual pathway and hippocampus; (iv) the frontal lobe to include dorsolateral prefrontal cortex, frontal pole, frontal eye fields and supplementary eye fields; (v) subcortical regions including the thalamus and basal ganglia. Where studies did not provide stereotaxic coordinates we used figures and text descriptions to categorise the activity into these regions.

### Parkinson’s disease

Visual hallucinations are the most common hallucination modality in Parkinson’s disease occurring in approximately 22–38 % of clinic-based samples [9]. The occurrence of visual hallucinations in patients with Parkinson’s disease is associated with worse outcome, including nursing home placement [10], increased morbidity and mortality [11] and cognitive decline [12, 13]. Typically, visual hallucinations are of animals, people or objects [14], begin in the second half of the disease, are persistent and get progressively worse [15].

#### Methodological considerations

As cognitive decline and visual hallucinations are linked in Parkinson’s disease, cognition needs to be controlled for when comparing patients with and without visual hallucinations. Although hallucinations and illusions in the visual modality are more common than other senses [16], patients with visual hallucinations may also experience auditory hallucinations, delusions and lack insight. These symptoms are themselves associated with cortical changes so that a change identified in a given cortical area might not be linked specifically to visual hallucinations. We did not differentiate studies reporting Parkinson’s disease at different stages of cognitive decline (Parkinson’s disease and Parkinson’s disease dementia) but report studies of Dementia with Lewy bodies in a separate section.

#### Structural imaging findings

Ramirez-Ruiz et al. [17]—Parkinson’s disease patients with (*N* = 18) and without visual hallucinations (*N* = 20) and healthy controls (*N* = 21). They found grey matter volume loss bilaterally in lateral occipital regions and the parietal lobe when compared to patients without visual hallucinations and frontal regions when compared to healthy controls.

Ibarretxe-Bilbao et al. [18]—Three groups of patients with Parkinson’s disease: (i) with dementia (*N* = 9), (ii) without dementia but with visual hallucinations (*N* = 16) and (iii) without dementia or visual hallucinations (*N* = 19). Using a region of interest approach, they found volume differences in the hippocampus. Non-demented Parkinson’s disease patients with visual hallucinations showed hippocampal atrophy restricted to the hippocampal head compared to those without visual hallucinations. By contrast, the Parkinson’s disease dementia patients demonstrated diffuse hippocampal atrophy compared to those without dementia or visual hallucinations. The authors hypothesised that hippocampal involvement begins in the head, where it results in visual hallucinations, and then spreads to the tail where it results in additional memory impairment. To test this hypothesis, the same group performed a 30-month follow-up study (12 patients with Parkinson’s disease and visual hallucinations, 14 patients with Parkinson disease without visual hallucinations and 12 healthy controls) [19]. At follow-up, 9 of the 12 visual hallucinators had gone on to develop dementia. Comparing their baseline and follow-up scans, the visual hallucinators had greater grey matter loss in bilateral parietal cortex (predominantly precuneus and supra-marginal gyrus), insula, superior and inferior temporal gyrus, superior and inferior frontal gyrus, anterior and posterior cingulate gyrus, thalamus and limbic areas including hippocampus. Patients with Parkinson’s disease without visual hallucinations at baseline did not exhibit the same pattern of atrophy at follow-up and none had developed dementia.

Sanchez-Castaneda et al. [20]—Parkinson disease patients with dementia and visual hallucinations (*N* = 7) compared to those without hallucinations (*N* = 8). They found reduced grey matter in the left lateral orbitofrontal cortex.

Meppelink et al. [21]—Parkinson’s disease patients with visual hallucinations (*N* = 11) compared to those without hallucinations (*N* = 13). Although they identified grey matter reductions bilaterally in prefrontal and parietal cortices in both groups compared to healthy controls (*N* = 14), they did not find significant differences in grey matter between patients with and without visual hallucinations.

Watanabe et al. [22]—Parkinson’s disease without dementia with (*N* = 13) and without visual hallucinations (*N* = 13). Visual hallucinations were associated with reduced volume in bilateral occipital regions, right supramarginal gyrus and left fusiform gyrus, bilateral dorsolateral prefrontal cortex, frontal pole and the middle portion of the left cingulate gyrus.

Goldman et al. [23]—Parkinson’s disease with visual hallucinations (*N* = 25, 6 of whom also had auditory hallucinations) compared to Parkinson’s disease without visual hallucinations (*N* = 25). Within apriori regions of interest, they found that visual hallucinations were associated with reduced grey matter volume, bilaterally in the cuneus, fusiform gyrus, lateral occipital cortex, inferior parietal lobule, cingulate and precentral gyrus as well as regions in the right lingual gyrus and left paracentral gyrus.

Gama et al. [24]—Parkinson disease (*N* = 39) and healthy controls (*N* = 10). The Parkinson’s disease group was divided into visual hallucinators (*N* = 11) and non-hallucinators (*N* = 28). Using a region of interest approach, they found that visual hallucinators had reduced grey matter volume in the left opercular frontal gyrus and left superior frontal gyrus compared to healthy controls.

### Dementia with Lewy bodies

Dementia with Lewy bodies accounts for up to 30.5 % of all dementia cases [25]. Visual hallucinations are one of the core features of Dementia with Lewy bodies along with fluctuating consciousness and a movement disorder [25–27]. The prevalence of visual hallucinations in patients with Dementia with Lewy bodies may be as high as 80 %, typically of people or animals.

#### Methodological considerations

The high prevalence of visual hallucinations and their inclusion in the diagnostic criteria mean that it is difficult to compare patients with and without visual hallucinations in this condition. Most studies have compared patients with Dementia with Lewy bodies to another condition, for example, Alzheimer’s disease, while matching for cognitive impairment. Where visual hallucinations are not the primary research question, patient hallucination status may not be reported. As in Parkinson’s disease, patients with Dementia with Lewy bodies may experience hallucinations in other modalities, delusions and lack of insight. Cortical differences identified in studies of Dementia with Lewy bodies may thus be attributable to visual hallucinations, associated symptoms or the clinical condition used as control.

#### Structural imaging findings

Sanchez-Castaneda et al. [20]—Dementia with Lewy bodies with visual hallucinations (*N* = 6) compared to Dementia with Lewy bodies without visual hallucinations (*N* = 6). Using a region of interest analysis, they found that patients with Dementia with Lewy bodies and visual hallucinations had reduced grey matter volume in the right inferior frontal gyrus. They also found an association between the severity of visual hallucinations in Dementia with Lewy bodies and grey matter volume reduction in the right inferior frontal gyrus and left precuneus. Occipital, parietal and temporal lobe regions were included in the analysis but were not found to be significantly different between the two groups.

Middlekoop et al. [28]—Dementia with Lewy bodies with (*N* = 20) and without (*N* = 3) visual hallucinations. They did not find significant differences in occipital lobe volume (controlling for whole-brain volume) in an analysis of variance (ANOVA) model combining patients with Alzheimer’s disease (see below). The trend difference between Dementia with Lewy bodies patients with and without visual hallucinations was in the opposite direction to that reported in other studies with a larger occipital volume in hallucinators than non-hallucinators.

Beyer et al. [29]—Dementia with Lewy bodies with unspecified visual hallucination status (*N* = 18) compared to Parkinson’s disease dementia with unspecified visual hallucination status (*N* = 15). Cortical atrophy was more pronounced in the Dementia with Lewy bodies group in occipital, temporal (including hippocampus and amygdala) and parietal cortices.

Josephs et al. [30]—Patients with posterior cerebral atrophy and visual hallucinations (*N* = 7 scanned) compared to those without visual hallucinations (*N* = 7 scanned) and healthy controls (*N* = 38). Compared to controls, bilateral grey matter atrophy was found in the basal forebrain, thalamus, hypothalamus and basal ganglia in visual hallucinators. These changes were not found in the group without visual hallucinations compared to controls. Both groups with and without visual hallucinations had atrophy in the primary visual cortex and midbrain compared to controls; however, the atrophy was more pronounced in the visual hallucinator group. Including patients that did not participate in the scanning study, 10 of 13 patients with visual hallucinations had features of parkinsonism, 6 had myoclonus and 8 had a rapid eye movement sleep disorder. At follow-up (>2 years), all 13 patients in this group met criteria for a diagnosis of Dementia with Lewy Bodies.

Whitwell et al. [31]—Dementia with Lewy bodies (*N* = 72, 62.5 % with visual hallucinations) compared to matched Alzheimer’s patients (*N* = 72) and normal controls (*N* = 72). Compared to normal controls, grey matter loss was identified in the dorsal midbrain, posterior hippocampus, insula, frontal and parietal lobes and surrounding the third ventricle. No regions were more atrophied in the Dementia with Lewy bodies group than the Alzheimer’s disease group on whole-brain analysis; however, a pre-defined region of interest in the dorsal midbrain had greater atrophy in the Dementia with Lewy bodies group.

Delli Pizzi et al. [32]—Dementia with Lewy bodies and visual hallucinations (*N* = 18) compared to Alzheimer’s disease without hallucinations (*N* = 15) and age-matched healthy controls (*N* = 14). Dementia with Lewy bodies was associated with reduced cortical thickness bilaterally in the pericalcarine cortex, lingual gyrus, cuneus, precuneus and superior parietal gyrus. The cortical thinning in the right hemisphere precuneus and superior parietal gyrus was correlated with hallucination severity.

### Alzheimer’s disease

Visual hallucinations are the most common hallucination modality in patients with Alzheimer’s disease with an estimated prevalence of 13 % [33]. Alongside other psychotic features in Alzheimer’s disease, they precipitate admission to psychiatric hospitals and nursing home placement [34]. As with Parkinson’s disease, they are linked to more rapid cognitive decline and worse overall prognosis [35]. In Alzheimer’s disease, patients typically hallucinate people, animals, insects and objects [36].

#### Methodological issues

As for Parkinson’s disease and Dementia with Lewy bodies, visual hallucinations are the commonest modality hallucinated. However, as in these conditions, visual hallucinations co-occur with other modalities, delusions and loss of insight and are associated with greater cognitive decline, all of which may contribute to the cortical changes identified.

#### Structural imaging findings

Holroyd et al. [37]—Alzheimer’s disease with (*N* = 7) and without (*N* = 7) visual hallucinations, matched for cognitive score. They found that patients with visual hallucinations had significantly smaller occipital lobes, controlling for whole-brain volume.

Middlekoop et al. [28]—Alzheimer’s disease with (*N* = 3) and without (*N* = 22) visual hallucinations. They did not find significant differences in occipital lobe volume (controlling for whole-brain volume) in an ANOVA model which included the Dementia with Lewy bodies group described above. As with Dementia with Lewy bodies, the trend found was in the opposite direction to that expected, with larger occipital volume in hallucinators compared to non-hallucinators.

Donovan et al. [38]—A longitudinal study over 3 years of changes in cognition (Cohort *N* = 812, Alzheimer’s disease at baseline *N* = 188). Although the modality of hallucinations was not specified, they are likely to have been predominantly visual, based on previous prevalence studies. They found reduced lateral parietal cortical thickness predicted increasing hallucinations but not occipital (lingual gyrus), frontal (including dorsolateral prefrontal cortex), inferior temporal or superior parietal cortex.

### Schizophrenia

Unlike the conditions reviewed above, in schizophrenia, visual hallucinations are less prevalent than auditory verbal hallucinations. The weighted mean point prevalence of visual hallucinations in a recent meta-analysis was 27 % (range of 4–65 %) with that of auditory hallucinations 59 % (range 25–86 %) [39]. Patients with visual hallucinations invariably experience hallucinations in other modalities, either at the same time or on different occasions [40]. In schizophrenia, visual hallucinations are associated with poorer prognosis [41] and are typically people, faces, animals, objects or events (e.g. visions of fires) and frightening [39].

#### Methodological issues

The fact that visual hallucinations in schizophrenia never occur in isolation influences the choice of control group and the inferences that can be drawn. One approach is to use schizophrenia patients with auditory verbal hallucinations but without visual hallucinations. The disadvantage is that regions involved in both hallucination modalities will not be identified in this type of analysis. Another approach is to use patients with schizophrenia but without hallucinations. This will identify all regions associated with hallucinations but is unable to link a given cortical region with one or other modality. Comparison with healthy controls has the additional problem of confounding changes related to schizophrenia.

#### Structural imaging findings

Jardri et al. [7]—Adolescent patients with first episode psychosis, a prodromal state that may or may not progress to schizophrenia (*N* = 20). Compared to healthy controls, they found a decrease in cortical thickness in visual and auditory association cortex but not in primary visual or auditory cortex.

Amad et al. [42]—Two subgroups of patients with schizophrenia: (i) with auditory hallucinations (*N* = 17) and (ii) with auditory and visual hallucinations (*N* = 16). Using a region of interest analysis, they found that hippocampal mean volumes were increased in the auditory and visual hallucinator group, with bilateral hypertrophy of the anterior and posterior end of CA1 and subiculum. It was hypothesised that these changes were due to plastic modification of the hippocampus related to changes in hippocampal connectivity found in the same study.

Cachia et al. [43]—Same design as Amad et al. [42] using a mathematical descriptor of gyral architecture, an index of early cortical development. They reported decreased gyral folding in the right hemisphere as a whole in patients with auditory and visual hallucinations. Using a region of interest analysis, decreased gyral folding was also found in right superior parietal cortex and the left sylvian fissure.

### Charles Bonnet syndrome

Charles Bonnet syndrome describes the association of visual hallucinations and eye disease, occurring in ~10 % of people with eye disease [44, 45] (range 0.4 % [46]—63 % [47]). The main risk factor for developing Charles Bonnet Syndrome is a reduction in visual acuity [45]. The most common type of visual hallucination is simple and unformed followed by patterns, people, animals, faces, landscapes and objects [39, 48].

#### Methodological issues

Charles Bonnet Syndrome differs from the other conditions reviewed above in that, by definition, it is not associated with hallucinations in other modalities [49] or progressive cognitive decline [50]. However, unlike other studies, the impact of visual loss on the visual cortex needs to be taken into account. As yet there are no structural imaging studies comparing patients with and without visual hallucinations in eye disease.

### Summary of imaging findings

Table 1 summarises the evidence presented above. Occipital atrophy is found in each clinical condition within sub-regions of visual association cortex in the lateral, dorsal and ventral occipital lobe. It remains unclear whether the occipital cortex is diffusely affected in all conditions or whether the distribution of atrophy varies from one condition to another. Similarly, there is insufficient detail to specify whether the primary visual cortex is affected in each condition. Notable exceptions to the finding of occipital atrophy are Meppelink et al. [21] in Parkinson’s disease and Middlekoop et al. [28] in Dementia with Lewy bodies and Alzheimer’s disease. As noted above, the Middlekoop et al. finding is based on small numbers. The negative finding reported by Meppelink et al. may relate to a conservative whole-brain analysis of the data. Although an absence of difference is also reported in a region of interest analysis of the left fusiform gyrus, no mention is made of whether this is also the case in other occipital regions.

**Table 1:** Cortical changes associated with visual hallucinations

Brain region	Clinical condition			
	Schizophrenia	Alzheimer’s disease	Dementia with Lewy bodies and PCA	Parkinson’s disease and Parkinson’s disease dementia
**Occipital lobe**				
Primary visual cortex			Atrophy [30, 32]	Atrophy [17, 22]
Secondary visual cortex	Atrophy [7]^a^		Atrophy [32]	Atrophy [17, 22, 23]
Gross occipital lobe		Atrophy [37]		
**Parietal lobe**				
Superior Lobule	Reduced Gyri [43]		Atrophy [32]	Atrophy [17]
Inferior Lobule	Atrophy [7]^a^	Atrophy [38]		Atrophy [19, 22, 23]
**Temporal lobe**				
Hippocampus			Atrophy [31]^a^	
CA1	Hypertrophy [42]			
Subiculum	Hypertrophy [42]			
Head				Atrophy [18]
Middle fusiform gyri				Atrophy [22, 23]
**Frontal lobe**				
Pre-central cortex			Atrophy [20]	Atrophy [19, 23]
Dorsolateral prefrontal cortex			Atrophy [20]	Atrophy [22, 24]
Frontal pole				Atrophy [19, 20, 22, 24]
Middle Cingulate cortex				Atrophy [23]
**Subcortical structures**				
Thalamus			Atrophy [30]	Atrophy [19]
Basal ganglia			Atrophy [30]	

^a^Comparison with healthy controls

The parietal and ventral temporal lobe also contains regions considered part of the extended visual system. As in the occipital lobe, parietal regions were found atrophied in patients with visual hallucinations across all conditions, in particular the inferior parietal lobe. Atrophy was identified here in Parkinson’s disease, Dementia with Lewy bodies and Alzheimer’s disease literature and, although not specifically mentioned in the text, the figure illustrating visual association cortex atrophy in first episode psychosis includes the region [7], with evidence of changes in gyrification in schizophrenia [43]. Atrophy is also reported in the superior parietal lobule, but there is insufficient evidence to determine whether this varies across conditions. The ventral extension of the visual system into the temporal lobe is affected in Parkinson’s disease in some studies but was reported as unaffected in the left fusiform gyrus by Meppelink et al. [21]. It was not identified in the study of Dementia with Lewy bodies by Sanchez-Castaneda et al. [20] using a conservative whole-brain analysis and fell outside the region of interest studied by Middlekoop et al. [28]. Ventral temporal regions of interest were not associated with increases in hallucinations of unspecified modality in Alzheimer’s disease [38] and were not mentioned or illustrated in the first episode psychosis study [7]. It therefore remains unclear whether ventral temporal cortex is implicated in visual hallucinations across all conditions.

Regions of frontal atrophy were identified in Parkinson’s disease and Dementia with Lewy bodies. The regions cluster around areas linked to attentional networks whose dysregulation has been proposed as a mechanism for visual hallucinations [51], overlapping those involved in eye movements (medial and lateral frontal eye fields in the pre-central cortex, supplementary frontal eye fields in the middle portion of the cingulate gyrus [52] ). Frontal regions of interest were not associated with hallucinations of unspecified modality in Alzheimer’s disease [38] and were not mentioned or illustrated in the first episode psychosis study [7]. Like ventral temporal regions, it remains unclear whether frontal regions are implicated in all conditions associated with visual hallucinations.

Changes in the hippocampus have been noted in both Parkinson’s disease and schizophrenia but in opposite directions. In schizophrenia, relative hypertrophy was found compared to patients with auditory hallucinations (visual + auditory hallucinator > auditory hallucinator). In Parkinson’s disease, hippocampal atrophy was found (visual hallucinator < non-hallucinator). The region has not been reported as a separate region of interest in the Alzheimer’s disease or Dementia with Lewy bodies literature so it remains unclear whether changes in hippocampal volume also occur in these conditions.

The thalamus was not reported as affected in any of the studies reviewed using a whole-brain analysis or included as a separate region of interest. It was noted as an area of atrophy at 2–3 year follow-up in patients with Parkinson’s disease and visual hallucinations, which was not found to the same degree in Parkinson’s disease patients without visual hallucinations [19]. It was also found in patients with Dementia with Lewy bodies compared to controls [30].

## Discussion

Our review set out to examine whether the same neurophysiological mechanism predisposes to visual hallucinations in all conditions by comparing the distribution of cortical changes. To date, no imaging studies have investigated visual hallucinations from a trans-diagnostic perspective, necessitating a comparison of studies using very different methodological approaches. Despite this limitation, patterns of cortical change common to different clinical conditions seem to emerge. We discuss below what the findings reveal about the pathophysiology of susceptibility to visual hallucinations and the wider clinical significance of the findings for the development of effective treatments.

### Methodological considerations

It is perhaps surprising that a degree of consistency has been found between studies of different conditions, given that many factors will contribute to their variability. Beyond technical details of the scanners or image processing and segmentation techniques used, there are important differences in analysis strategy. These include the choice of control for a given condition, the covariates accounted for in the analysis (for example the presence and severity of dementia, age and additional symptoms such as hallucinations in other modalities, delusions, movement disorder, fluctuation in cognition, etc.), whether the study investigates pre-specified regions or the whole brain and whether regions associated with visual hallucinations are identified by a categorical approach (comparing groups with and without visual hallucinations) or by correlating cortical volume with the severity or frequency of hallucinations. Furthermore, studies vary in the way hallucination status is defined. Most use the response to a questionnaire item or set of items within widely used instruments [e.g. neuropsychiatry inventory (NPI), positive and negative syndrome scale (PANSS), scale of assessment of positive symptoms (SAPS), unified Parkinson’s disease rating scale (UPDRS)] or structured clinical interviews. As such, the time window of what defines visual hallucination or non-hallucination status may vary between studies, ranging from occurrence in the preceding weeks to the entire course of the illness. Similarly, a distinction may or may not be drawn between visual hallucinations and other visual phenomena such as illusions/pareidolia that are associated with hallucination susceptibility [53]. Such methodological differences make it difficult to draw firm conclusions about differences in patterns of atrophy between conditions but add to the likelihood that any similarities identified are robust and valid.

### The cortical signature of visual hallucination susceptibility

de Morsier argued that all visual hallucinations were linked to the visual thalamo-cortical system. As might be predicted by this account, changes in visual cortex were associated with visual hallucination susceptibility in all conditions. The parietal lobe, a region de Morsier held of particular significance because of its connections to the pulvinar, was also implicated. A key piece of missing evidence is whether these cortical regions are also affected in patients with Charles Bonnet Syndrome which, if correct, would add support to de Morsier’s claim. What de Morsier had not predicted is that regions outside the visual thalamo-cortical system in the frontal lobe and hippocampus are also linked to visual hallucinations. Current models of visual hallucinations in Parkinson’s disease and Dementia with Lewy bodies focus on abnormal interactions between top-down attentional processing (derived from frontal systems) and bottom-up visual perceptual processing (from occipital systems) [51, 54]. The atrophy within frontal executive/attentional regions and visual networks in Parkinson’s disease and Dementia with Lewy bodies is consistent with this view. However, there is an absence of evidence as to whether the same areas are also implicated in Alzheimer disease, schizophrenia and Charles Bonnet Syndrome. Similarly, the hippocampus, although not playing a prominent role in current theories of visual hallucinations, is easily accommodated based on its connectivity to the visual system [55] and might account for hallucinations of familiar or remembered experience in Dementia with Lewy bodies, Parkinson’s disease and schizophrenia. As for frontal regions, it is unclear whether the hippocampus is affected in all conditions.

### The functional neurophysiology of aberrant cortical activation

**Fig. 2:**
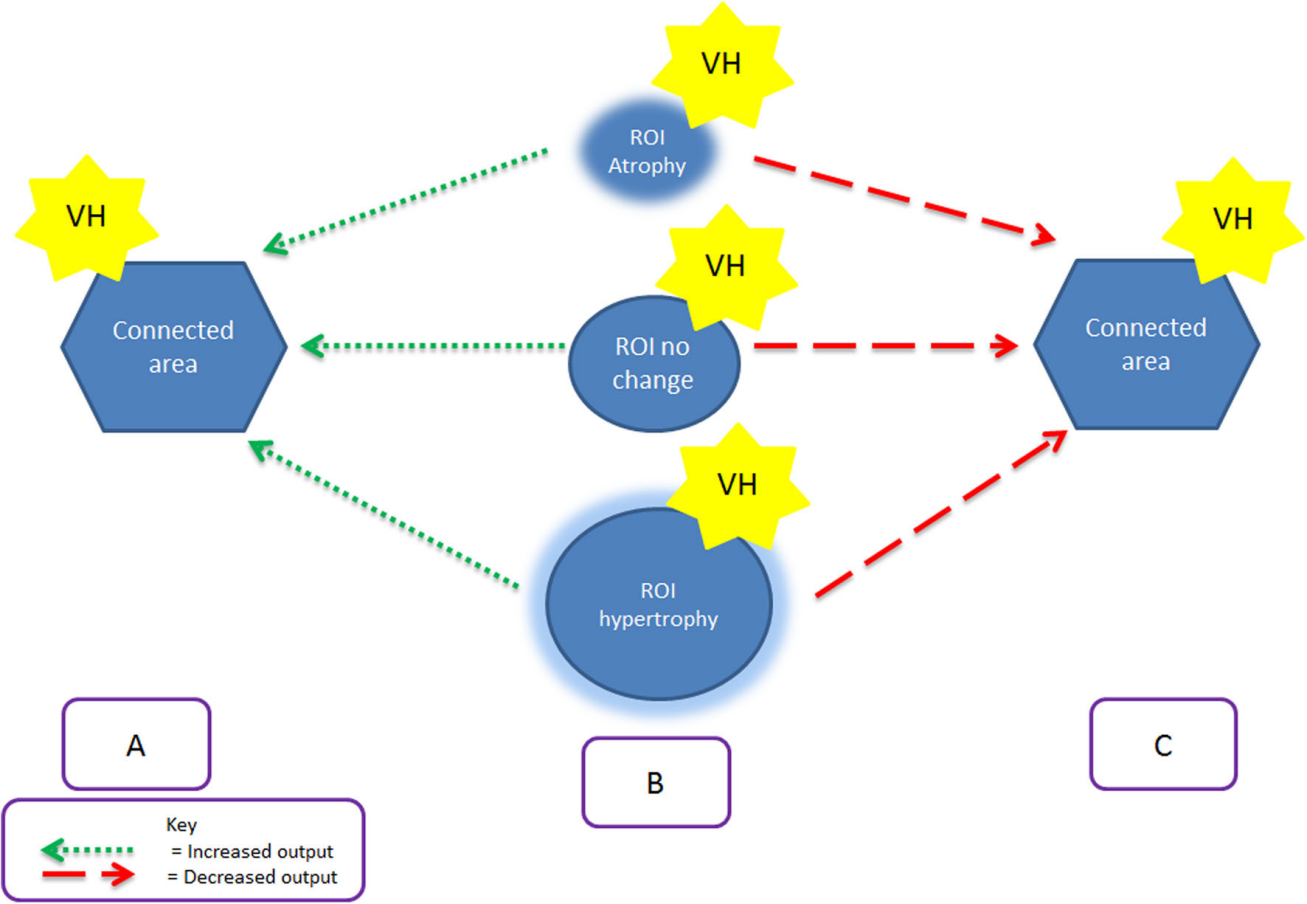
A summary of the theoretical mechanisms by which changes in cortical volume within a region of interest (ROI) are linked to increased cortical excitability and visual hallucinations (VH). **a** Increased excitatory output from region of interest to connecting area results in visual hallucinations in connected area. The increased output may be associated with atrophied (*upper row*), normal volume (*middle row*) or increased volume (*lower row*) cortex. **b** Increased excitation within the region of interest results in visual hallucinations. The increased excitation may be associated with atrophied (*upper row*), normal volume (*middle row*) or increased volume (*lower row*) cortex. **c** Decreased output from region of interest to connecting area (cortical de-afferentation) results in loss of control/modulation leading to excitability and visual hallucinations in connected area. The decreased output may be associated with atrophied (*upper row*), normal volume (*middle row*) or increased volume (*lower row*) cortex

Functional imaging evidence of spontaneous visual cortical activation at the time of a visual hallucination [5, 6] leaves open the question of what causes it. The evidence reviewed here suggests regions where spontaneous activations have been identified are atrophied in patients susceptible to visual hallucinations. Atrophy is traditionally interpreted as causing a deficit in function. However, if reflecting changes in internal cortical architecture, for example, a relative loss of intra-cortical inhibitory neurones, it could result in hyper-excitability. This may be restricted to the area of atrophy or be propagated to connected areas. Alternatively, atrophy may result in a loss of output/function to another part of the network resulting in a deficit of modulatory control (cortical de-afferentation) and consequent excitation and hyper-excitability within the area to which it connects. These three conceptual mechanisms could apply without detectable cortical atrophy or even with an increase in cortical volume (see Fig. 2). Changes in white matter might also result in cortical hyper-excitability and visual hallucinations through related mechanisms [56] but have not been included in the figure.

Our finding of occipital and parietal atrophy in patients with visual hallucinations, irrespective of condition, is likely to reflect mechanism B with regions of atrophy marking local hyper-excitability. The distribution of hyper-excitable cortex will define the type of visual hallucination experienced by a given patient [5] and, if varying systematically across conditions, would account for variations in hallucination content in different clinical contexts. In contrast, the frontal regions found atrophied are unlikely to be directly responsible for the content of visual hallucinations. These areas are more likely to represent mechanism C, reflecting loss of top-down control over visual cortices or mechanism A, reflecting activty propagated across the network from the frontal lobe. The hippocampal hypertrophy found in schizophrenia and atrophy found in Parkinson’s disease might both reflect the same mechanism: localised hyper-excitability (B), altered hippocampal influence on visual cortex (C) or propagated activity from hippocampus (A). Of note, the hippocampus showed spontaneous increases in activity at the time of visual hallucinations in a patient with schizophrenia [6].

### Clinical implications

de Morsier recognised that visual hallucinations occurred in a wide range of clinical conditions; however, the true extent of their prevalence and associated clinical problems are only recently appreciated. Visual hallucinations are associated with poorer prognosis, cognitive decline, distress and the move from independent living to a care setting in many of the conditions reviewed. There is a need to better understand how to treat the symptom and prevent such outcomes, but there is a current lack of evidence. Understanding whether the cortical changes predisposing to visual hallucinations are the same or different across conditions will help design future treatment trials. If the mechanism predisposing to visual hallucinations is always the same, one treatment may be effective for all clinical conditions. Alternatively, if there are several distinct mechanisms that predispose to visual hallucinations, a different type of treatment may be required for each mechanism, with individualised combinations of treatment for patients whose visual hallucinations are caused by more than one mechanism.

## Conclusion

de Morsier’s clinical intuition that visual hallucinations are linked to aberrant activity within visual networks seems to be correct. The evidence reviewed here points to occipital and parietal atrophy in patients susceptible to visual hallucinations, irrespective of clinical context. Whether frontal and hippocampal regions are also included in this network or specific to a subset of conditions remains unclear. Like de Morsier, we continue to lack techniques to determine whether all visual hallucinations share a common neurophysiology. However, the indirect evidence of cortical changes predisposing to visual hallucinations reveals features common to all conditions that help inform the ongoing search for effective treatment.
